# Association between triglyceride-glucose index and bone mineral density in US adults: a cross sectional study

**DOI:** 10.1186/s13018-023-04275-6

**Published:** 2023-10-30

**Authors:** Huixia Zhan, Xinyu Liu, Shenghua Piao, Xianglu Rong, Jiao Guo

**Affiliations:** 1 Science and Technology Innovation Center, Guangzhou University of Chinese Medicine, Guangzhou, China; 2 Guangdong Metabolic Diseases Research Center of Integrated Chinese and Western Medicine, Guangzhou, China; 3 Key Laboratory of Glucolipid Metabolic Disorder, Ministry of Education of China, Guangzhou, China; 4 Guangdong TCM Key Laboratory for Metabolic Diseases, Guangzhou, China; 5 The Institute of Chinese Medicinal Sciences, Guangdong Pharmaceutical University, Guangzhou, China; 6 The First Affiliated Hospital of Guangdong Pharmaceutical University, Guangzhou, China

**Keywords:** Triglyceride–glucose index, Bone mineral density, Osteoporosis, NHANES

## Abstract

**Objective:**

Disorders in glucose and lipid metabolism have been shown to exert an influence on bone metabolism. The TyG index, which combines measures of glucose and triglycerides, provides insights into the overall metabolic status. However, the investigation of concurrent disturbances in glucose and lipid metabolism and their specific implications for bone metabolism remains limited in the existing research literature. This study aimed to explore the correlation between the TyG index and bone mineral density (BMD) in US adults.

**Methods:**

In the National Health and Nutrition Examination Survey (NHANES), subjects were classified based on the TyG index into four groups (< 7.97, 7.97–8.39, 8.39–8.85, > 8.86). Linear regression analysis was conducted to determine the *β* value and 95% confidence interval (CI). Four multivariable models were constructed. Restricted cubic spline analyses and piecewise linear regression were employed to identify the association between the BMD and TyG index. An analysis of subgroups was also conducted in this study.

**Results:**

Significant variations in related characteristics were found among the US adult population, who were distributed into four groups based on the quartiles of the TyG index. A negative correlation between the TyG index and lumbar spine BMD was observed. In the multi-adjusted models, compared to Q1 of the TyG index, the *β* for Q4 of the TyG index for lumbar spine BMD was [*β* = − 0.008, 95% CI (− 0.017, 0)] in US adults. The association between the TyG index and lumbar spine BMD was found to be nonlinear (all nonlinear *p* < 0.001), with a threshold value based on restricted cubic spline analyses. Above the threshold point, the *β* for lumbar spine BMD was − 0.042 (95% CI, − 0.059, − 0.024). Below the threshold points, no significant difference was observed (*p* > 0.05). No significant interactions were observed among subgroups based on age, gender, presence of diabetes, BMI, and use of antidiabetic and antihyperlipidemic agents. Similar patterns of association were observed in total and subtotal bone density.

**Conclusions:**

This study identified a nonlinear association between the TyG index and BMD in the US population. Furthermore, an increased level of the TyG index may indicate a higher risk of osteoporosis among US adults. These findings highlight the importance of considering glucose and lipid metabolism disturbances in understanding bone health and the potential for developing preventive strategies for osteoporosis.

**Supplementary Information:**

The online version contains supplementary material available at 10.1186/s13018-023-04275-6.

## Introduction

Osteoporosis is a chronic metabolic bone disease manifested by a reduction in bone mass and degradation of microarchitectural bone tissue [1]. This condition arises from an imbalance in the activities of osteoclasts and osteoblasts, resulting in a decrease in bone mineral density and irreversible loss of bone mass. This loss occurs due to accelerated bone resorption and/or delayed bone formation [2]. The measurement of bone mineral density (BMD) is commonly employed as an indicator of bone health. DXA-derived BMD measurements obtained from the hip, lumbar spine, total, subtotal and femur neck are utilized as key assessment tools for diagnosing osteoporosis clinically. As life expectancy has increased, there has been a rise in the prevalence of osteoporosis, leading to a decline in quality of life and an increase in medical expenses. Apart from genetic factors, age, and lifestyle choices [3, 4], such as lipid or glucose metabolism [5–7], have recently drawn considerable attention due to their influence on bone metabolism.

Previous studies have highlighted the potential link between abnormal glycolipid metabolism and the development of osteoporosis. The association between type 2 diabetes mellitus (T2DM) and bone density has been investigated in several studies, resulting in varying conclusions. Some studies have shown lower bone density in individuals with T2DM; while, others have reported normal or even higher bone mineral density (BMD) [8, 9]. In a cross sectional study, it was found that elevated levels of total cholesterol (TC) and triglycerides (TG) are associated with an increased risk of osteoporosis [10]. However, there was no significant association found between low-density lipoprotein cholesterol (LDL-C) and high-density lipoprotein cholesterol (HDL-C) levels with osteoporosis. Notably, a study by Cui et al. did not find any significant association between HDL-C levels and BMD in both pre- and postmenopausal subjects [11]. On the other hand, Jeong et al. observed a positive correlation between HDL-C levels and BMD in postmenopausal Korean women [12]. However, it is common to observe coexisting disorders in glucose and lipid metabolism. The Triglyceride Glucose (TyG) index, which reflects the status of glucolipid metabolism as a composite indicator of glucose and triglycerides, has been found to be inversely correlated with femoral neck bone mineral density (BMD) among non-diabetic men aged 50 years and older, as well as postmenopausal women in the KNHANES 2008–2011 dataset [13]. Nevertheless, there is a lack of research investigating the relationship between glucolipid metabolism and bone density in the general population. Therefore, further studies are needed to gain a comprehensive understanding of the association between glucolipid metabolism and bone density in the general population.

This study enrolled adults aged 20 years and older and utilized data from the National Health and Nutrition Examination Survey (NHANES) database. The primary aim was to investigate the relationship between the TyG index and bone mineral density (BMD).

## Methods

### Data source and population study

The NHANES survey, conducted every two years since 1999, used a complex, stratified, multiple-stage probability sampling approach to examine the health and nutritional conditions of non-institutionalized individuals in the US. Approximately 5000 people were sampled in the NHANES survey each year, and data were released every two years. There were interviews consisting of demographic and socioeconomic inquiries, nutritional and health-related inquiries, as well as physical testing consisting of medical, dental, physiologic, and laboratory testing. These data provide investigators and the public with valuable insights into the diagnosis of disease, as they are available to access. Our data combine four cycles of NHANES carried out from 2011 to 2018.

**Fig. 1:**
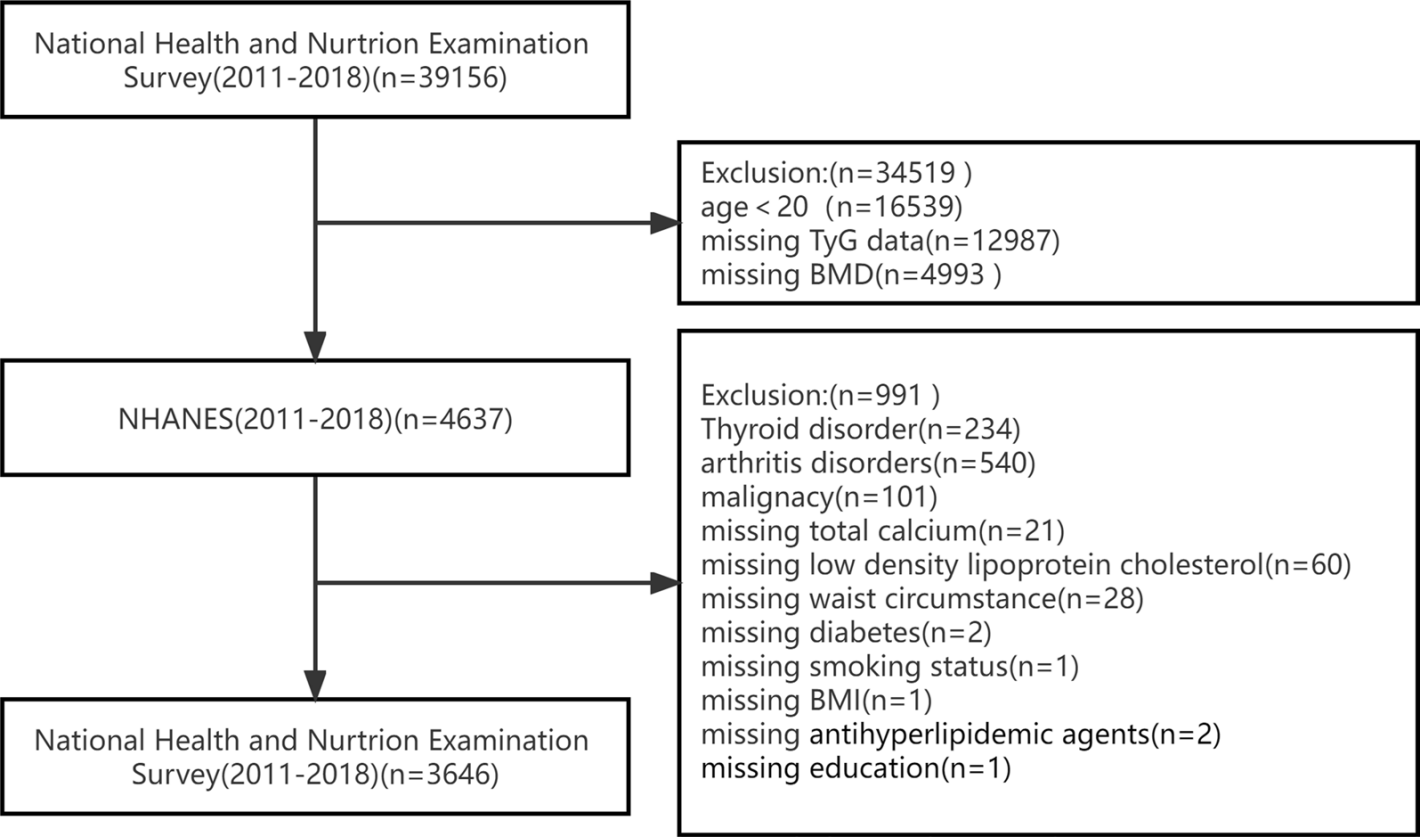
Flowchart of participants selection

Our investigation population (*n* = 39,156) was restricted to adults, aged ≥ 20 years (*n* = 22,617). We excluded instances with absent TyG information (*n* = 12,987), missing BMD information (*n* = 4,993), suffering from chronic and thyroid diseases affecting bone metabolism (*n* = 234), arthritis disease (*n* = 540) and cancer (*n* = 101). Additionally, subjects lacking covariables (*n* = 116) were excluded. Finally, 3646 individuals were screened and included (Fig. 1). All subjects signed a written consent form, and the research was authorized by the National Center for Health Statistics. Herein, the STROBE (Strengthening the Reporting of Observational Studies in Epidemiology) declaration was adhered to as the Helsinki Declaration.

### TyG index evaluation

The formula for calculating the TyG index is Ln [TG (mg/dL) fasting plasma glucose (FPG; mg/dL)/2] [14]. Morning blood samples were obtained upon fasting overnight to investigate the amounts of TG and glucose in the blood. An automatic biochemistry analyzer was used to measure the concentration of TG and FPG. A Roche Cobas 6000 chemistry analyzer and a Roche Modular P chemistry analyzer were used to determine the serum TG levels. A Roche/Hitachi Cobas C 501 chemistry analyzer was used to measure FPG using the hexokinase-mediated reaction.

### Outcome variable

BMD was assessed utilizing a Hologic QDR-4500A fan-beam densitometer and dual-energy X-ray absorption (DXA; Hologic, Inc., Bedford, Massachusetts), which are commonly used for assessing and treating osteoporosis. BMD was assessed using dual-energy X-ray absorption (DXA) images obtained using Apex 3.2 software and a Hologic Discovery model A densitometer (Hologic, Inc., Bedford, Massachusetts). All measurements were conducted by NHANES radiological technologists with extensive training and certification.

### Covariates

Based on the literature, the following covariates were included: age, sex, race and ethnicity, educational level, moderate recreational activities, serum uric acid, serum calcium, serum phosphorus, waist circumference (WC), body mass index (BMI), lumbar spine BMD, FPG, HDL-C, TC, LDL-C, TG, creatinine levels, 25-hydroxyvitamin D; In NHANES, information on self-reported race/ethnicity were derived from responses to survey questions on race and Hispanic ethnicity. we categorized the participants into the following 4 races and ethnicities: non-Hispanic white, non-Hispanic black, Mexican American, and Other race. Educational level was divided into 3 levels (high school or less, some college, and college graduate or higher). Diabetes (ever been told by a doctor you have diabetes, fasting glucose 126 mg/dL [to convert to millimoles per liter, multiply by 0.0555], or hemoglobin A1c [HbA1c] 6.5% [to convert to proportion of total hemoglobin, multiply by 0.01]). Smoking status was determined by the survey question, “Have you smoked at least 100 cigarettes in your entire life,” Participants who answered “yes” were defined as smokers. Moderate recreational activity (yes or no). Information regarding the acquisition of these covariates is available on the NHANES website (https://www.cdc.gov/nchs/nhanes/).

### Statistical analyses

The study presents the characteristics of the participants as means with 95% confidence intervals for continuous variables and percentage frequencies with 95% confidence intervals for categorical variables. The statistical analysis involved t tests for continuous data and χ2 tests for categorical data. The multiple linear regression analysis was conducted to assess the independent link between TyG index and lumbar spine BMD. We built 4 models: model 1, adjusting for no covariates. Model 2, adjusting for age and gender. Model 3, age, gender, race, education, moderate recreational activities, diabetes, uric acid, total calcium, phosphorus, WC, LDL-C, 25-hydroxyvitamin D(25OHD. Model 4, model3 plus antihyperlipidemic agents. Restricted cubic spline curves were utilized to evaluate potential nonlinear associations between TyG index levels and BMD. Stratified analyses by subgroup variables were presented with a fully adjusted Model 4. Log-likelihood ratio test was used to assess the interaction effects between TyG index and subgroup variable. We analyzed the data using R program packages (http://www.R-project.org, R Foundation) and Free Statistics (vs. 1.7) [15]. A *P*-value of 0.05 was determined statistically significant.

## Results

### Characteristics of study population

Herein, 3646 subjects aged ≥ 20 years were included in the study. Based on their baseline characteristics (Table 1), participants were categorized into quartiles according to the TyG index. The average age of the subjects was 37.4 ± 11.2 years, with 53.6% were male. As the TyG index increased, age, BMI, WC, uric acid levels, TC levels, LDL-C levels, TG levels, and FPG levels increased. Conversely, HDL-C levels decreased with the increasing TyG index. Subjects in the high TyG group (Q4) exhibited higher values for creatinine levels. They also had higher rates of males and smoking addicts compared to the other groups (Q1–Q3). When compared to participants in the low TyG group (Q1-Q3), those in the high TyG group (Q4) had lower values for HDL-C and 25-hydroxyvitamin D (25OHD) levels.

**Table 1:** Characteristics according to triglyceride-glucose index quartile

Variables	Total (*n* = 3646)	Q1 6.19–7.97 (*n* = 911)	Q2 7.97–8.39 (*n* = 912)	Q3 8.39–8.85 (*n* = 911)	Q4 8.86–10.95 (*n* = 912)	*p*
Age, years	37.4 ± 11.2	33.8 ± 10.5	36.0 ± 11.3	38.6 ± 11.2	41.1 ± 10.4	< 0.001
Gender, %						< 0.001
Male	1956 (53.6)	343 (37.7)	465 (51)	521 (57.2)	627 (68.8)	
Female	1690 (46.4)	568 (62.3)	447 (49)	390 (42.8)	285 (31.2)	
BMI, kg/cm^2^	28.1 ± 6.5	25.8 ± 5.9	27.2 ± 6.6	29.1 ± 6.3	30.3 ± 6.1	< 0.001
WC, cm	95.3 ± 15.8	87.7 ± 13.9	92.8 ± 15.9	98.3 ± 15.1	102.5 ± 14.4	< 0.001
Education, %						< 0.001
Less than high school	666 (18.3)	121 (13.3)	155 (17)	176 (19.3)	214 (23.5)	
High school	784 (21.5)	182 (20)	182 (20)	200 (22)	220 (24.1)	
More than high school	2196 (60.2)	608 (66.7)	575 (63)	535 (58.7)	478 (52.4)	
Race, %						< 0.001
Non-Hispanic white	1185 (41.2)	282 (39)	303 (42)	314 (42.8)	286 (40.8)	
Non-Hispanic black	723 (25.1)	267 (36.9)	210 (29.1)	135 (18.4)	111 (15.8)	
Mexican American	580 (20.1)	93 (12.8)	122 (16.9)	173 (23.6)	192 (27.4)	
Other race	391 (13.6)	82 (11.3)	86 (11.9)	111 (15.1)	112 (16)	
Diabetes, %						< 0.001
Yes	205 (5.6)	10 (1.1)	22 (2.4)	36 (4)	137 (15)	
No	3441 (94.4)	901 (98.9)	890 (97.6)	875 (96)	775 (85)	
Moderate recreational activities, %						0.002
Yes	1667 (45.7)	450 (49.4)	436 (47.8)	407 (44.7)	374 (41)	
No	1979 (54.3)	461 (50.6)	476 (52.2)	504 (55.3)	538 (59)	
Smoking status, %						< 0.001
Yes	1339 (36.7)	261 (28.6)	323 (35.4)	347 (38.1)	408 (44.7)	
No	2307 (63.3)	650 (71.4)	589 (64.6)	564 (61.9)	504 (55.3)	
Uric_acid, mg/dL	5.4 ± 1.4	4.8 ± 1.1	5.2 ± 1.3	5.6 ± 1.3	5.9 ± 1.4	< 0.001
Creatinine, mg/dL	72.5 (61.0, 84.0)	69.0 (59.2, 81.3)	73.4 (61.9, 84.2)	72.5 (61.0, 84.9)	74.3 (61.9, 85.8)	< 0.001
Total calcium, mg/dL	9.3 ± 0.3	9.3 ± 0.3	9.3 ± 0.3	9.3 ± 0.3	9.3 ± 0.3	0.018
Phosphorus, mg/dL	3.7 ± 0.6	3.7 ± 0.5	3.7 ± 0.6	3.6 ± 0.6	3.6 ± 0.5	< 0.001
TC, mg/dL	187.4 ± 38.4	167.5 ± 31.7	181.0 ± 33.3	193.3 ± 36.0	207.6 ± 40.2	< 0.001
HDL_C, mg/dL	53.0 ± 14.7	62.0 ± 15.1	55.9 ± 14.0	50.8 ± 12.4	43.5 ± 10.3	< 0.001
TG, Mean ± SD	107.0 ± 65.2	46.8 ± 10.8	75.2 ± 11.0	110.9 ± 18.3	195.1 ± 63.4	< 0.001
LDL, mg/dL	112.9 ± 34.1	96.2 ± 27.9	110.0 ± 30.0	120.3 ± 32.5	125.1 ± 37.9	< 0.001
FPG, mg/dL	104.0 ± 31.1	93.0 ± 8.6	97.2 ± 9.8	101.8 ± 15.3	124.0 ± 53.8	< 0.001
25(OH)D, nmol/l	58.7 ± 23.3	58.6 ± 24.6	59.3 ± 24.1	58.8 ± 22.1	58.3 ± 22.2	0.819
Lumbar spine BMD, g/cm^2^	1.0 ± 0.1	1.0 ± 0.1	1.0 ± 0.1	1.0 ± 0.1	1.0 ± 0.1	< 0.001
Total BMD, g/cm^2^	1.1 ± 0.1	1.1 ± 0.1	1.1 ± 0.1	1.1 ± 0.1	1.1 ± 0.1	0.006
Subtotal BMD, g/cm^2^	1.0 ± 0.1	1.0 ± 0.1	1.0 ± 0.1	1.0 ± 0.1	1.0 ± 0.1	< 0.001
Antihyperlipidemic agents, *n* (%)						< 0.001
Yes	224 (6.1)	15 (1.6)	50 (5.5)	51 (5.6)	108 (11.8)	
No	2385 (65.4)	666 (73.1)	589 (64.6)	588 (64.5)	542 (59.4)	
Other	1037 (28.4)	230 (25.2)	273 (29.9)	272 (29.9)	262 (28.7)	
Antidiabetic agents, *n* (%)						< 0.001
Yes	178 (4.9)	11 (1.2)	16 (1.8)	32 (3.5)	119 (13)	
No	2385 (65.4)	666 (73.1)	589 (64.6)	588 (64.5)	542 (59.4)	
Other	1083 (29.7)	234 (25.7)	307 (33.7)	291 (31.9)	251 (27.5)	

Data were mean ± SD or median (IQR) for skewed variables or numbers (proportions) for categorical variables

*BMI* Body mass index, *WC* Waist circumference, *TyG* Triglyceride-glucose index, *TC* Total cholesterol, *FPG* Fasting plasma glucose, *TG* Triglycerides, *HDL-C* High-density lipoprotein cholesterol, *LDL-C* Low-density lipoprotein cholesterol, *HbA1c* Glycated hemoglobin, *BMD* Bone mineral density, *TyG* Triglyceride-glucose index, *IQR* Interquartile range

### Correlation between TyG index and BMD

**Fig. 2:**
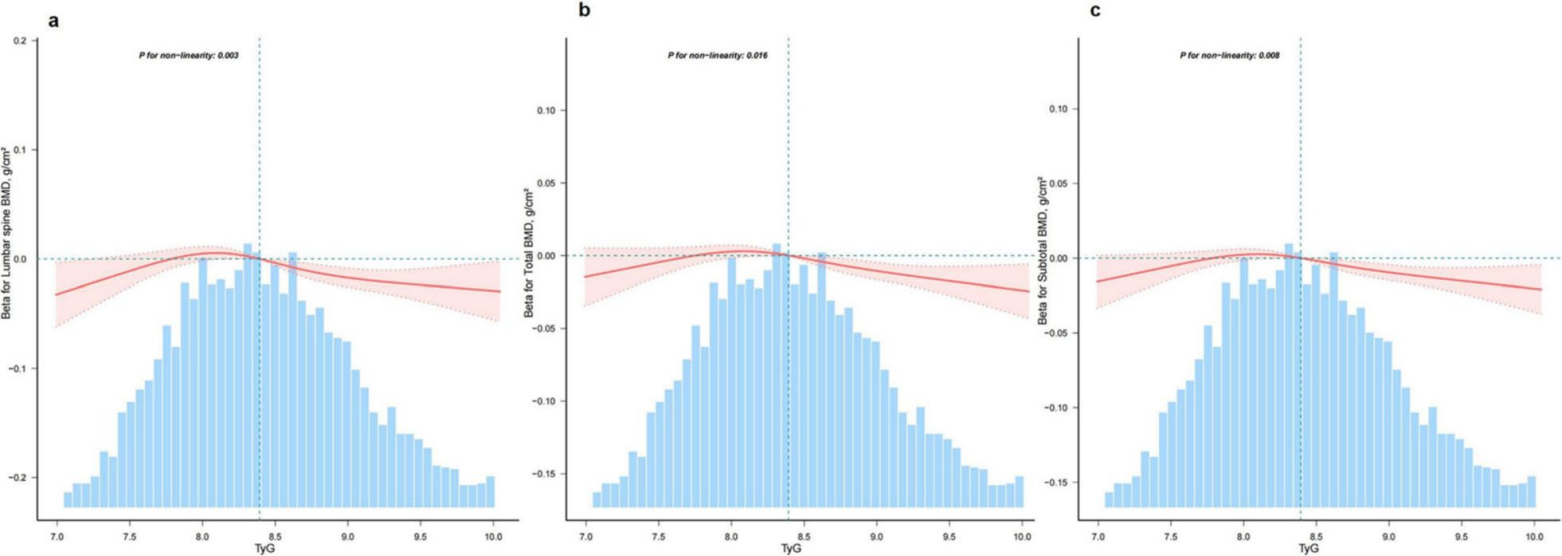
Smooth curve fitting of relationship between TyG index with bone mineral density. Association between TyG index with bone mineral density. Solid and dashed lines represent the predicted value and 95% confidence intervals. They were Adjust for age, gender, race, education, moderate recreational activities, diabetes, uric acid, total calcium, serum phosphorus, waist circumference, ldl-c, smoke status, 25-hydroxyvitamin D(25OHD), antihyperlipidemic agents. Only 99% of the data is displayed

Our study utilized multivariate linear regression analysis to evaluate the correlation between the TyG index and bone mineral density (BMD) in four distinct models. The unadjusted model revealed a negative correlation between the TyG index and lumbar spine BMD [− 0.028 (− 0.035, − 0.02), Table 2]. This negative correlation persisted even after adjusting for potential confounding factors [− 0.008 (− 0.017, 0), Table 2)]. Additionally, a sensitivity analysis using the TyG index as categorical variables (quartiles) yielded consistent results, particularly in the highest quartile group. Furthermore, we discovered a nonlinear relationship (*p* < 0.05 for nonlinearity) between the TyG index and BMD after accounting for potential confounders such as age, race, gender, education, moderate recreational activities, diabetes, uric acid, total calcium, serum phosphorus, waist circumference, ldl-c, smoke status, 25-hydroxyvitamin D, and lipid-lowering drugs (Fig. 2). This nonlinearity was statistically significant, indicating a threshold value for BMD. Below this threshold point, we observed no significant association between the TyG index and BMD. However, once the TyG index exceeded the threshold point, we observed a decrease in BMD (Table 3). The same trend was also found in total and subtotal bone density (Tables 2 and 3).

**Table 2:** Association between triglyceride-glucose index and bone mineral density among 3646 US adults

Variables	Model 1	Model 2	Model 3	Model 4
	*β* (%95 CI)	*β* (%95 CI)	*β* (%95 CI)	*β* (%95 CI)
*Lumbar bone bmd*				
TyG	− 0.028 (− 0.035 ~ − 0.02)	− 0.024 (− 0.031 ~ − 0.016)	− 0.009 (− 0.017 ~ 0)	− 0.008 (− 0.017 ~ 0)
Q1	0(Ref)	0(Ref)	0(Ref)	0(Ref)
Q2	0 (− 0.013 ~ 0.013)	0.002 (− 0.011 ~ 0.016)	0.013 (0 ~ 0.026)	0.014 (0.001 ~ 0.027)
Q3	− 0.022 (− 0.036 ~ − 0.009)	− 0.018 (− 0.032 ~ − 0.005)	0.005 (− 0.009 ~ 0.019)	0.006 (− 0.008 ~ 0.02)
Q4	− 0.051 (− 0.064 ~ − 0.038)	− 0.045 (− 0.059 ~ − 0.031)	− 0.019 (− 0.034 ~ − 0.003)	− 0.017 (− 0.033 ~ − 0.002)
*P* for trend	< 0.001	< 0.001	0.0096	0.0142
*Total bmd*				
TyG	− 0.002 (− 0.007 ~ 0.004)	− 0.01 (− 0.016 ~ − 0.005)	− 0.007 (− 0.013 ~ − 0.001)	− 0.007 (− 0.012 ~ − 0.001)
Q1	0(Ref)	0(Ref)	0(Ref)	0(Ref)
Q2	0.007 (− 0.002 ~ 0.017)	0 (− 0.009 ~ 0.009)	0.002 (− 0.006 ~ 0.011)	0.003 (− 0.005 ~ 0.012)
Q3	0.008 (− 0.002 ~ 0.018)	− 0.002 (− 0.011 ~ 0.008)	0.004 (− 0.005 ~ 0.014)	0.005 (− 0.004 ~ 0.015)
Q4	− 0.007 (− 0.017 ~ 0.002)	− 0.023 (− 0.033 ~ − 0.013)	− 0.017 (− 0.027 ~ − 0.007)	− 0.015 (− 0.026 ~ − 0.005)
*P* for trend	0.1732	< 0.001	0.0039	0.007
*Subtotal bmd*				
TyG	0.009 (0.004 ~ 0.015)	− 0.005 (− 0.01 ~ − 0.001)	− 0.006 (− 0.011 ~ − 0.001)	− 0.005 (− 0.011 ~ 0)
Q1	0(Ref)	0(Ref)	0(Ref)	0(Ref)
Q2	0.016 (0.006 ~ 0.025)	0.003 (− 0.005 ~ 0.011)	0.003 (− 0.005 ~ 0.011)	0.004 (− 0.004 ~ 0.012)
Q3	0.021 (0.011 ~ 0.03)	0.004 (− 0.005 ~ 0.012)	0.006 (− 0.003 ~ 0.014)	0.006 (− 0.002 ~ 0.015)
Q4	0.012 (0.002 ~ 0.022)	− 0.015 (− 0.024 ~ − 0.006)	− 0.014 (− 0.023 ~ − 0.005)	− 0.013 (− 0.023 ~ − 0.004)
*P* for trend	0.0081	0.002	0.0054	0.009

Model 1 was not adjusted. Model 2 was adjusted for age and gender. Model 3 was adjusted for all covariables in model 2 plus race education, moderate recreational activities, diabetes, uric acid, total calcium, serum phosphorus, waist circumference, low-density lipoprotein cholesterol, smoke status, 25-hydroxyvitamin D(25OHD). Model 4 was adjusted for all covariables in model 3 plus antihyperlipidemic agents

*TyG* Triglyceride-glucose index, *CI* Confidence interval, *Ref* Reference

**Table 3:** Threshold effect analysis of relationship of TyG index with bone mineral density

	Adjusted *β*(95% CI)	*p*
One-line linear regression model	− 0.009 (− 0.017 ~ 0)	< 0.001
Lumbar spine BMD		
*Two-piecewise linear regression model*		
TyG < 8.161	0.017 (− 0.015, 0.049)	0.14
TyG ≥ 8.161	− 0.042 (− 0.059, − 0.024)	< 0.001
Likelihood ratio test		< 0.001
Total BMD		
*Two-piecewise linear regression model*		
TyG < 8.359	0.008 (− 0.008, 0.024)	0.14
TyG ≥ 8.359	− 0.027 (− 0.042, − 0.013)	< 0.001
Likelihood ratio test		< 0.001
Subtotal BMD		
*Two-piecewise linear regression model*		
TyG < 8.406	0.005 (− 0.009, 0.018)	0.14
TyG ≥ 8.406	− 0.032 (− 0.045, − 0.018)	< 0.001
Likelihood ratio test		< 0.001

Adjust for age, gender, race, education, moderate recreational activities, diabetes, uric acid, total calcium, serum phosphorus, waist circumference, low-density lipoprotein cholesterol, smoke status, 25-hydroxyvitamin D(25OHD), antihyperlipidemic agents

### Subgroup analysis

**Fig. 3:**
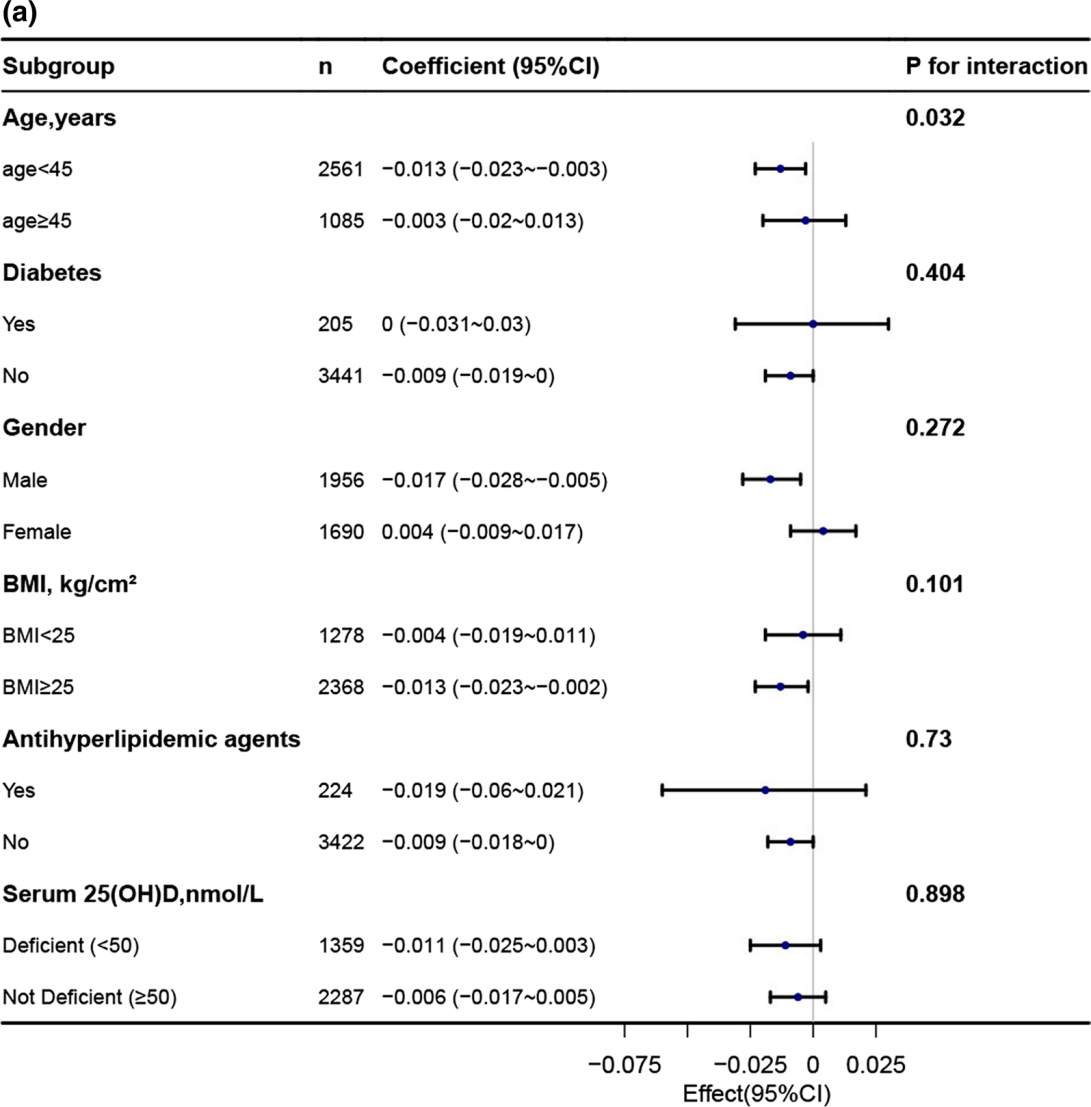

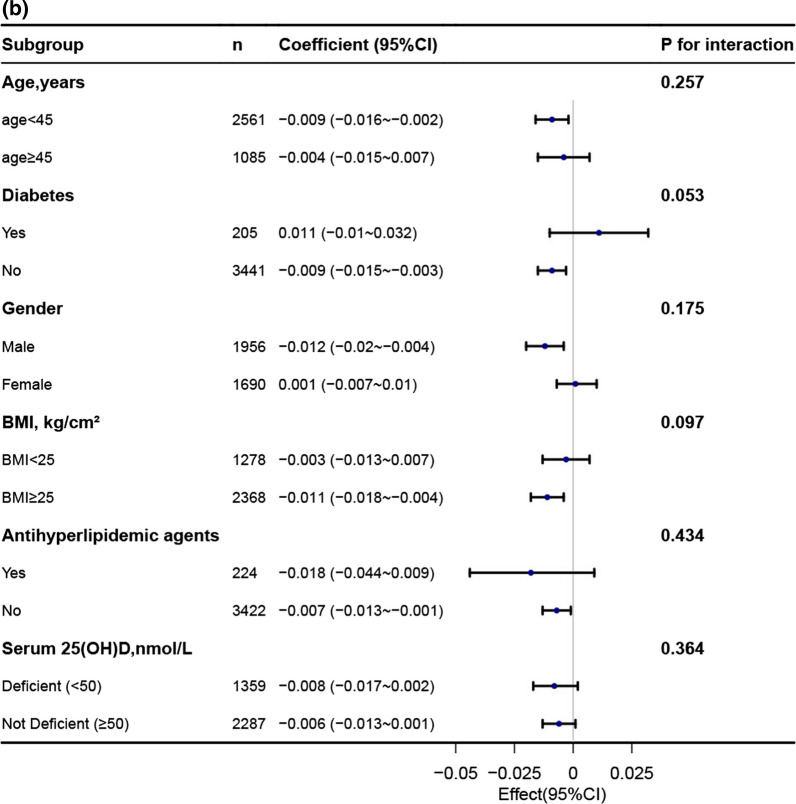

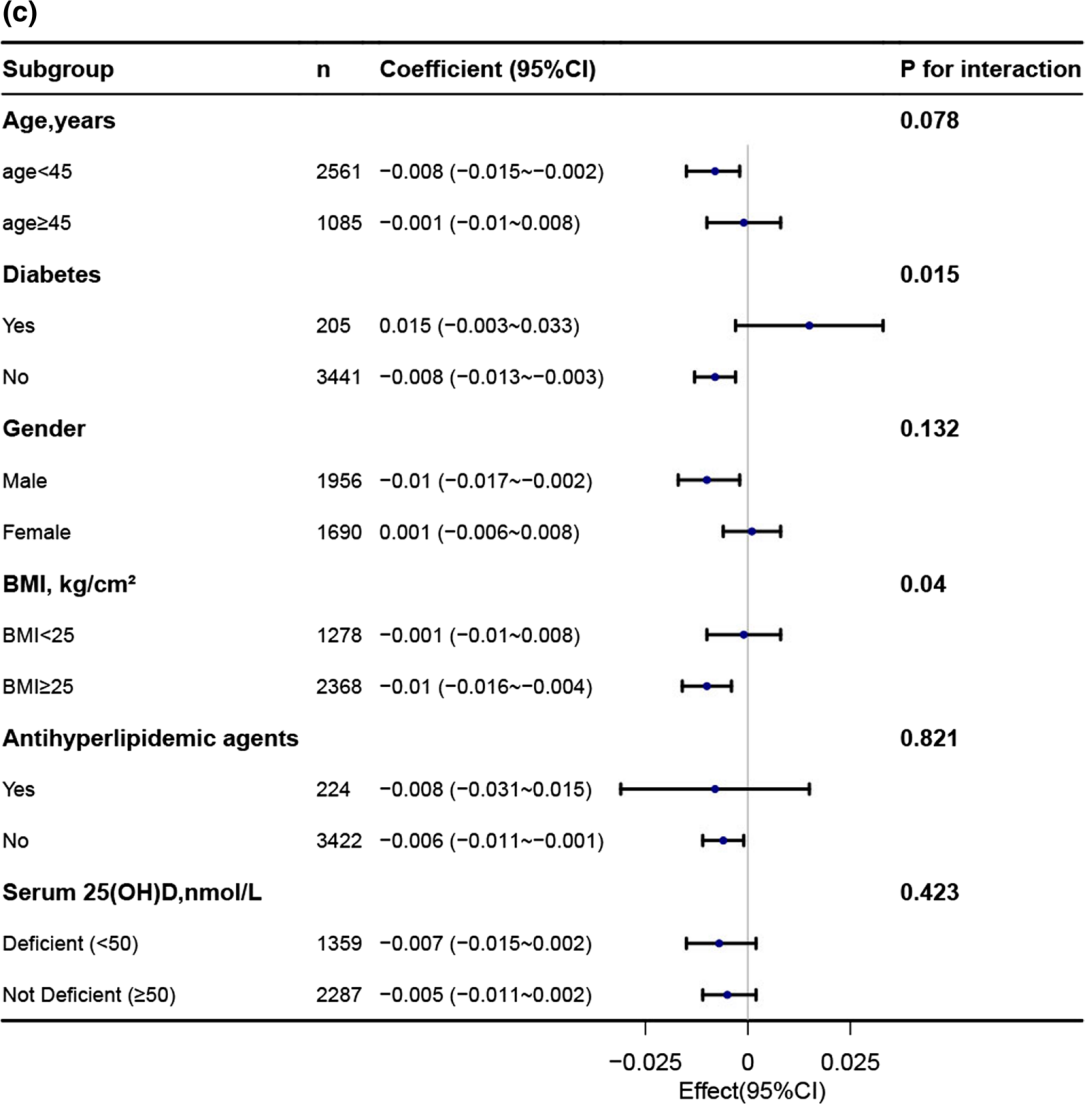
Association between TyG index with bone mineral density. **a** Association between TyG index with lumbar spine bone mineral density. **b** Association between TyG index with total bone mineral density. **c** Association between TyG index with subtotal bone mineral density

In order to explore potential effect modifications on the relationship between the TyG index and BMD, we performed stratified analyses in several subgroups. These subgroups included age, gender, diabetes, BMI, 25-hydroxyvitamin D(25OHD) and antihyperlipidemic agents. However, we did not observe any significant interactions in any of these subgroups (Fig. 3, Additional file 1: Table 1). It is worth noting that although the *p*-value for the interaction of age was less than 0.05, it may not be statistically significant when considering multiple testing (Additional file 1: Table 1).

Each stratification was adjusted for age, gender, race, education, moderate recreational activities, diabetes, uric acid, total calcium, serum phosphorus, waist circumference, ldl-c, smoke status, 25-hydroxyvitamin D(25OHD), antihyperlipidemic agents except the stratification factor itself.

## Discussion

This large cross sectional study of American adults revealed a negative correlation between the TyG index and BMD. Additionally, an inverted U-shaped relationship was observed between the TyG index and BMD among US adults. Both stratified and sensitivity analyses confirmed the robustness of the association between the TyG index and bone mineral density.

Recently, the incidence of osteoporosis has been steadily increasing, presenting significant challenges for both individuals and society [16]. It is important to note that osteoporosis is influenced by various factors, including age [17], gender [18], endocrine disorders [19], metabolic disorders [20], as well as lipid and glucose metabolism [6, 7]. The TyG index, which is calculated based on FPG and TG levels, serves as an indicator of glucolipid metabolism and has been considered as a valuable marker for cardiovascular disease [21]. However, there is currently limited and inconclusive clinical evidence regarding the association between the TyG index and bone mineral density in the general population.

The relationship between glucose metabolism and bone mineral density (BMD) is still not well understood, and studies in different populations have yielded varied results. Poorly controlled type 2 diabetes patients have lower levels of 25-hydroxyvitamin D (25OHD) and osteocalcin (OC), suggesting glycemic control's independent role in low BMD [22]. Higher hemoglobin levels also protect against osteoporosis in older men with type 2 diabetes [23]. There has been a negative correlation observed between TyG-BMI and BTMs in individuals with type 2 diabetes mellitus (T2DM), suggesting that higher TyG-BMI values may be associated with impaired bone turnover [24]. In the NHANES 2005–2018 data, Liu et al. found that in participants with abnormal glucose metabolism, abnormal glucose levels correlate with higher BMD [8]. Recent observational studies strengthen the association between type 2 diabetes and hip fracture risk [25], despite individuals with type 2 diabetes exhibiting higher BMD [26]. Mitchell et al.'s [27] findings indicate that higher fasting glucose levels are linked to smaller bone area at the hip but possibly greater BMD, challenging the notion that elevated glucose levels are generally detrimental to bone health. Further research is needed to confirm these findings and understand the underlying mechanisms. In conclusion, the relationship between glucose metabolism and BMD is complex, varied across populations, and warrants further investigation for a comprehensive understanding.

The association between serum lipid levels and bone mineral density (BMD) has been examined in different populations, yielding varied results. In postmenopausal Chinese women, a nonlinear relationship was found between TC, LDL-C, HDL-C and lumbar spine BMD [28]. Additionally, higher levels of TC and TG were associated with an increased risk of osteoporosis [10]. Another study showed that elevated HDL-C levels were independent risk factors for bone loss in both men and women [7]. Among young Northeast Indian women, low levels of HDL-C and TG, and high levels of homocysteine (Hcy), were linked to osteopenia and osteoporosis [29]. Serum triglycerides were inversely associated with BMD, potentially influenced by vitamin D status in older adults [30]. Interestingly, a U-shaped curve was found between triglycerides and total lumbar BMD, suggesting a threshold effect [31]. Furthermore, a study demonstrated an inverse correlation between HDL-C levels and BMD [32]. In the NHANES 2011–2018 data, a study conducted by Xiao et al. found a negative relationship between LDL-C levels and lumbar BMD in the young- and middle-aged population [5]. Additionally, Wang's study, after accounting for all relevant covariates, revealed an interesting inverted U-shaped curve representing the correlation between HDL-C levels and total BMD among adolescents aged 16–19 [33]. Conversely, in the general population aged 20–59, HDL-C levels demonstrated a positive association with lumbar BMD [34]. These findings suggest that lipid metabolism plays a role in bone health, but variations exist across populations and further research is needed to fully understand the complex relationship between lipid levels and BMD.

Research on the association between simultaneous disruptions in glucose and lipid metabolism and bone density is relatively scarce, with few studies conducted in the past. The study reveals that the TyG index has an inverse correlation with neck bone mineral density (BMD) in non-diabetic men aged 50 years and above, as well as postmenopausal women. The negative impact of insulin resistance on femoral neck BMD is particularly pronounced in women with a BMI below 23 kg/m^2^, indicating the influence of skeletal site, sex, and BMI on this relationship [13]. Among non-diabetic middle-aged and elderly individuals in China, the combination of the TyG index and BMI shows a positive association with bone mineral density (BMD) and bone geometry [35]. Furthermore, this combination is linked to a reduced risk of fractures. Significantly, the TyG index is significantly associated with a higher risk of low bone mass and osteoporosis. This highlights the vulnerability of individuals with insulin resistance to these conditions. Therefore, close monitoring of bone mass in insulin-resistant individuals and early implementation of preventive strategies are crucial in mitigating the occurrence of low bone mass and osteoporosis. In summary, the association between TyG index and BMD is complex and varies across skeletal sites, sex, and BMI. Continued research efforts will further enhance our understanding of the intricate relationship between Glucolipid Metabolism and bone health, leading to improved prevention and treatment strategies for optimizing bone health. Those inconsistent findings could be partially due to different population. Moreover, some important confounding factors, such as medications for diabetes, medications for diabetes, 25-hydroxyvitamin D are not considered in those studies. In the present study, we found negative associations between TyG index and BMD among 3646 US adults and high TyG index (> 8.855) were significantly associated with higher risk of osteoporosis after adjusting for potential confounders. In addition, our study revealed a nonlinear relationship between the TyG index and bone density. Above a specific threshold, the TyG index demonstrated a negative correlation with bone density.

The association between glucose and lipid metabolism with bone density can be explained by several mechanisms. Since bone plays a significant role in weight-bearing and metabolic activity, increasing age may impact bone mineral density (BMD) in individuals. Bone mass reaches its peak between the ages of 30 and 40, followed by a gradual decline [36]. Consequently, the loss of bone mass during middle age can contribute to the development of osteoporosis in later life, especially in women. To assess the role of age and gender in the correlation between the TyG index and BMD, we compared the results before and after adjusting for age and gender (model 1 vs. model 2). The relationship remained stable even after accounting for age and gender. Abnormalities in lipid metabolism, such as elevated triglyceride levels and reduced LDL cholesterol, can impact bone cell function and bone metabolism. After adjusting for plasma levels of LDL cholesterol and other relevant variables (model 3), the negative correlation between TyG index and BMD remained significantly unchanged (model 2). Additionally, Glucolipid Metabolism abnormalities may contribute to other metabolic changes, such as impaired calcium absorption and vitamin D metabolism [37], affecting bone density. In our study, the inclusion of calcium, phosphorus, and 25(OH) vitamin D, along with adjusting for the confounding factors, resulted in robust results. Furthermore, it should be noted that antihyperlipidemic agents and antidiabetic agents also influence bone density [38–40]. In our study, we took into account the use of these medications and found that the results remained consistently stable even after accounting for their effects. However, further research is needed to fully understand the complexities of these mechanisms and their interactions. Glucolipid Metabolism affects bone metabolism by decreasing bone formation and disrupting the balance of bone remodeling. Inflammatory response, triggered by Glucolipid Metabolism, can interfere with bone metabolism, leading to reduced bone formation and increased bone resorption. In this study, we observed a nonlinear relationship between the TyG index and BMD. Furthermore, we conducted threshold effect analysis and identified the cut-point value, which represents a major strength of our research.

Our study also has some limitations. Firstly, despite performing regression models, stratified analyses, and sensitivity analysis, there is still a possibility of residual confounding from unmeasured or unknown factors. Secondly, the current findings were based on a survey of adults in the United States, and generalizing them to other populations requires further investigation. Thirdly, due to the inherent limitations of cross sectional studies, we cannot establish a causal relationship between the TyG index and BMD. Therefore, it is important to validate these findings through future longitudinal studies.

## Conclusion

In conclusion, a nonlinear relationship exists between the TyG index with bone mineral density among the US population. Increased level of TyG index may indicate a higher risk of osteoporosis among US adults. Intervention studies are needed to determine whether improving the TyG index can enhance bone mineral density in the long run.

### Supplementary Information

**Additional file 1:** Subgroup analysis between triglyceride-glucose index and bone mineral density.

## Data Availability

The datasets analyzed during the current study are available in the website of the NHANES: https://www.cdc.gov/nchs/index.htm.

## Abbreviations

NHANES: National Health, and Nutrition Examination Survey
DXA: Dual-energy X-ray absorptiometry
IR: Insulin resistance
NAFLD: Non-alcoholic fatty liver disease
FPG: Fasting plasma glucose
BMI: Body mass index
WC: Waist circumference
TyG: Triglyceride-glucose index
TC: Total cholesterol
TG: Triglycerides
HDL-C: High-density lipoprotein cholesterol
LDL-C: Low-density lipoprotein cholesterol
HbA1c: Glycated hemoglobin
BMD: Bone mineral density
IQR: Interquartile range
CI: Confidence interval
Ref: Reference
