# Supplementary material for: Association between triglyceride-glucose index and bone mineral density in US adults: a cross sectional study
Source: J Orthop Surg Res. 2023 Oct 30;18:810. doi: 10.1186/s13018-023-04275-6 (PMC10614394; doi:10.1186/s13018-023-04275-6)
Supplement: Supplementary file 1 — Additional file 1: Subgroup analysis between triglyceride-glucose index and bone mineral density. [file 13018_2023_4275_MOESM1_ESM.docx]

***Supplementary Table 1.*** *Subgroup analysis between triglyceride-glucose index and bone mineral density*

|  | | **lubmar spine BMD** | | | **total BMD** | | **subtotal BMD** | |
| --- | --- | --- | --- | --- | --- | --- | --- | --- |
| **Subgroup analysis** | | ***β*（95%CI）*p* value** | | ***p* for interaction** | ***β*（95%CI）*p* value** | ***p* for interaction** | ***β*（95%CI）*p* value** | ***p* for interaction** |
| **age, years** | |  | | 0.032 |  | 0.257 |  | 0.078 |
| age＜45 | | -0.013 (-0.023~-0.003)** | |  | -0.009 (-0.016~-0.002)** |  | -0.008 (-0.015~-0.002)** |  |
| age≥45 | | -0.003 (-0.02~0.013) | |  | -0.004 (-0.015~0.007) |  | -0.001 (-0.01~0.008) |  |
| **Gender** | |  | | 0.272 |  | 0.175 |  | 0.132 |
| Male | | -0.017 (-0.028~-0.005)** | |  | -0.012 (-0.02~-0.004)** |  | -0.01 (-0.017~-0.002)** |  |
| Female | | 0.004 (-0.009~0.017) | |  | 0.001 (-0.007~0.01) |  | 0.001 (-0.006~0.008) |  |
| **Diabetes** | |  | | 0.404 |  | 0.053 |  | 0.015 |
| Yes | | 0 (-0.031~0.03) | |  | 0.011 (-0.01~0.032) |  | 0.015 (-0.003~0.033) |  |
| No | | -0.009 (-0.019~0) | |  | -0.009 (-0.015~-0.003)** |  | -0.008 (-0.013~-0.003)** |  |
| **BMI** | |  | 0.101 | |  | 0.097 |  | 0.04 |
| BMI＜25 | | -0.004 (-0.019~0.011) |  | | -0.003 (-0.013~0.007) |  | -0.001 (-0.01~0.008) |  |
| BMI≥25 | -0.013 (-0.023~-0.002)** | |  | | -0.011 (-0.018~-0.004)** |  | -0.01 (-0.016~-0.004)** |  |
| **Serum 25(OH)D,nmol/L** |  | | 0.898 | |  | 0.364 |  | 0.423 |
| Deficient (＜50） | -0.011 (-0.025~0.003) | |  | | -0.008 (-0.017~0.002) |  | -0.007 (-0.015~0.002) |  |
| Not Deficient (≥50） | -0.006 (-0.017~0.005) | |  | | -0.006 (-0.013~0.001) |  | -0.005 (-0.011~0.002) |  |
| **antihyperlipidemic agents** |  | | 0.73 | |  | 0.434 |  | 0.821 |
| Yes | -0.019 (-0.06~0.021) | |  | | -0.018 (-0.044~0.009) |  | -0.008 (-0.032~0.015) |  |
| No | -0.009 (-0.018~0) | |  | | -0.007 (-0.013~-0.001)** |  | -0.006 (-0.011~-0.001)** |  |

All stratification adjusted for age, gender, race, education, moderate recreational activities, diabetes, uric acid, total calcium, serum phosphorus, waist circumference, low-density lipoprotein cholesterol, smoke status, 25-hydroxyvitamin D(25OHD), antihyperlipidemic agents.

***P*＜0.01
